# A mixed-method study on adolescents’ well-being during the COVID-19 syndemic emergency

**DOI:** 10.1038/s41598-022-24007-w

**Published:** 2023-01-17

**Authors:** Alessandro Pepe, Eleonora Farina

**Affiliations:** 1 “R.Massa” Department of Human Sciences for Education, University of Milano-Bicocca, Milan, Italy; 2 LAB300, University of Milano-Bicocca, Milan, Italy

**Keywords:** Psychology, Health care

## Abstract

In this study, we set out to investigate adolescents’ levels of perceived well-being and to map how they went about caring for their well-being during the COVID-19 syndemic. Participants were 229 Italian adolescent high school students (48.9% males, mean age = 16.64). The research design was based on an exploratory, parallel, mixed-method approach. A multi-method, student-centered, computer-assisted, semi-structured online interview was used as the data gathering tool, including both a standardized quantitative questionnaire on perceived well-being and an open-ended question about how adolescents were taking charge of their well-being during the COVID-19 health emergency. Main findings reveal general low levels of perceived well-being during the syndemic, especially in girls and in older adolescents. Higher levels of well-being are associated with more affiliative strategies (we-ness/togetherness) whereas low levels of well-being are linked with more individualistic strategies (I-ness/separatedness) in facing the health emergency. These findings identify access to social support as a strategy for coping with situational stress and raise reflection on the importance of balancing the need for physical distancing to protect from infection, and the need for social closeness to maintain good mental health.

## Introduction

Officially declared a pandemic on 11 March 2020, the COVID-19 outbreak has resulted so far in over 550million cases and 6.3million deaths worldwide^1^. Although it appeared at the start of the pandemic that all populations around the world would be affected to the same extent, we now know that this is not the case. Indeed, while multiple sources initially claimed that "we were all in the same boat," two years after the onset of the pandemic, we are now in a position to state that "We are all on the same sea, but the boats from which we are dealing with the effects of COVID-19 are very different." Not only have the more strictly medical aspects differentially affected different populations (with the outcome of exacerbating inequalities), but the measures implemented by various governments for reducing the spread of the disease (e.g., lockdowns, restrictions on movement, school closures, and the adoption of distance education) have differentially affected different segments of the population in each country^2^.

### Covid-19 from a syndemic perspective

Variability in the evidence reported in the literature regarding the effects of the COVID-19 pandemic on different populations of interest and in different contexts may be explained by drawing on the concept of syndemic^3^. A syndemic is a situation in which two or more health conditions co-occur in environments of aggravated adversity and interact synergistically to yield worse health outcomes than each affliction would likely generate on its own^4^. Limiting the harms caused by COVID-19 will require paying far greater attention to so-called noncommunicable diseases (NCDs) and socioeconomic inequality than has been done up to now. Although NCDs have conventionally been analyzed in relation to the risk factors for cardiovascular diseases, cancers, chronic pulmonary diseases, and diabetes, scholars have recently emphasized^5^ that cardiovascular diseases, diabetes, cancer, and respiratory diseases frequently co-occur with both common mental disorders (such as depression and anxiety) and severe mental illnesses (such as schizophrenia and bipolar disorder). A *syndemic* is more than the outcome of a *pandemic* in terms of comorbidities; rather, it is an intertwining of biological and social conditions that increases an individual's susceptibility to harm or worsens their health outcomes. The most important implication of viewing the COVID-19 outbreak as a syndemic is that this helps to focus on its social origins. The vulnerability of younger and older citizens, ethnic minority communities, and key workers, who are frequently underpaid and enjoy less social welfare protection, points to an unacknowledged truth: no matter how effective a treatment or protective a vaccine, any exclusively biomedical solution for COVID-19 will fail.

### Impact of syndemic on adolescents’ well-being and mental health

The international scientific literature presents extensive research on the effects of the syndemic on individual well-being in different age groups and based on different methods of inquiry^6^. Adolescents, although at lower risk of death or severe illness due to COVID-19 than the adult population, are still having to cope at different levels with the negative impact of the public health emergency on their mental health^7^. Among other manifestations, the literature highlights anxiety-related, depressive, psychosomatic symptoms, as well as high levels of post-traumatic stress; these symptoms are more marked in girls, older adolescents, and adolescents with pre-existing vulnerabilities^8^. A recent review of 156 studies on changes in adolescents’ mental health during the COVID-19 emergency showed that outcomes had significantly worsened in several areas^9^. Among studies of depression, some 79% of studies found that participants’ symptoms had worsened, while 76% of studies of anxiety identified a worsening of symptoms, especially in girls and young women. Similarly, 70% of the research on stress and distress observed a clear increase in these phenomena with respect to the pre-pandemic period. Also considered in the review were studies—both longitudinal and cross-sectional—that found changes in subjective well-being, quality of life, and life satisfaction: the vast majority of authors identified a worsening of these dimensions. On the other hand, contrary to fears that adolescents would engage in greater substance abuse during the COVID-19 health emergency, findings regarding the use of substances have been mixed. A recent report by the U.S. National Institutes of Health identified a sharp decline in adolescent substance use in 2021^10^. The syndemic’s impact on substance use has likely been moderated by a number of factors, including changes in the social settings in which young people normally have the opportunity to use substances. In-person social interaction was drastically reduced in many contexts, thus reducing opportunities to drink alcohol^11^. In contrast, other studies showed that young people who remained more isolated during stay-at-home regimes used more cannabis than those who continued to socialize in person^12^.

However, in relation to mental health problems, contrasting results have been found both within and between categories. The overall decline in mental health is likely related to multiple factors implicated in the COVID-19 emergency internationally, including the specifics of different socioeconomic backgrounds^13^ as the concept of syndemic also reflects. Although most longitudinal studies on depression, anxiety and stress have documented an increase in symptoms over the period of the COVID-19 emergency, others have found no change or even a decrease in the incidence of these symptoms. Increases in suicide ideation and suicide attempts have been reported in several countries, but in one of the few studies conducted with a subgroup of marginalized youth, a significant reduction in episodes of self-harm was reported during the pandemic, potentially attributable to good service response^14^. Furthermore, different aspects of the syndemic setting likely generated different mental health problems. For example, a survey of U.S. students found that school-related concerns (e.g., lower quality online courses) were associated with increased depressive symptoms, while concerns related to home confinement per se (e.g., “cabin fever”) were associated with increased generalized anxiety symptoms^15^. It is also worth reporting the various studies during these syndemic years that also noted people's ability to detect positive aspects related to the emergency situation. For example, one line of research focused on the development of a form of wisdom derived from the ability to detect positive opportunities (such as spending more time with family members, developing forms of solidarity with other people) to develop new attitudes, behaviors, and values^16^. Other studies have pointed out that the emotional impact of syndemia, although predominantly characterized by emotions such as anxiety, sadness, and fear, over time also brings out positive emotions such as hope, trust, and tranquility^17^.

### Maintaining personal well-being during the syndemic

Studies that did not find significant changes in levels of well-being among youth have identified an association with the use of positive coping strategies in many cases^18,19^. Young people report that they used different ways to sustain resilience: in most cases, these strategies involved trying to maintain relational connections with significant others who were physically out of reach (friends and relatives), combined with more individual approaches to maintaining physical and mental well-being (exercising, spending time outdoors, meditating…).

Among the different factors that can reduce the risk of non-communicable disease during a syndemic, the literature recognizes coping strategies, along with perceived social support, as protective against the development of acute symptoms following exposure to particularly stressful events. Coping is the deployment of behavioral and cognitive strategies to modify negative aspects of one’s environment, and to minimize or escape internal threats induced by stress or trauma. Such strategies are diverse and can be more or less adaptive^20,21^. A more active coping style includes problem-oriented strategies, for example target the context as a means of solving difficulties, create an action plan, referring to someone, and be free to express and share feelings. Avoidance strategies include denial, substance use, and behavioral and mental detachment: trying to suppress emotions, withdrawing from people and enacting risky behaviors can be examples of avoidant coping style. Social support can be sought with a view to acquiring understanding or information or as an emotional outlet, which is a crucial resource to cope with stressful events and develop a positive attitude of acceptance, containment, and positive reinterpretation of events. In literature emerged that the use of avoidant coping strategies among adolescents was associated with overall higher levels of anxiety and depression and with other factors related to living conditions, such as having three or more siblings, having separated parents with low educational level^22^.

The restrictions imposed in the context of the COVID-19 health emergency have drastically reduced and disrupted access to many forms of social support, meaning that one coping strategy is less available or completely unavailable. However, studies show that family life during the initial severe lockdown of 2020, although it severely constrained adolescents’ drive for autonomy—hindering the fulfillment of a fundamental developmental task—acted as a key protective factor in their mental well-being^23,24^.

In light of the strong association between adolescents’ interactions with peers, friends, and family and their psychological well-being, it is of crucial importance to examine the factors that could further hinder or damage interpersonal interactions during this vulnerable stage of life.

### The present study

In this study, we set out to investigate adolescents’ levels of perceived well-being and to map how they went about caring for their well-being during the COVID-19 syndemic. In keeping with the literature reviewed above, we hypothesized that they would have drawn on both individual and social resources to feel good and safe, as well as making novel use—and possibilities to use—of indoor and outdoor spaces. We expected that different strategies would be associated with differential levels of well-being. More specifically, we hypothesized that a strategy of seeking to maintain satisfying and supportive relationships with family and peers would foster the deployment of more proactive coping attitudes and consequently higher levels of perceived well-being. In contrast, we predicted that more individualistic and inward-looking coping strategies would be associated with a tendency toward passivity, a diminished perception of being in control of the situation, and consequent lower levels of well-being.

## Method

### Sample

Participants were 229 Italian adolescent high school students. The sample was balanced in terms of gender, comprising 48.9% males (n = 112) and 48% females (n = 110); seven participants (3.1%) did not specify their gender. Participants’ ages ranged from 14 to 19 years (*M* = 16.64, *SD* = 1.46). The inclusion criteria were: (1) attending high school, (2) being aged between 14 and 19 years, (3) accepting the terms of participation in the research. We did not apply any exclusion criteria. We recruited a convenience sample via a non-probability sampling technique whereby participants are selected from the population only because they agree to participate^25^. We collected the data during the period from April 2021 to June 2021.

### Procedure and materials

This exploratory study was underpinned by a parallel mixed-method research design^26^ and its primary source of data was a multi-method, student-centered, computer-assisted web interview (CAWI)^27,28^. The research protocol comprised three main sections: (1) demographic background, (2) closed items about well-being, (3) two open-ended question about being an adolescent during the COVID-19 public health emergency (“At this time, what are the times, situations, or events that help you feel good?” and “If you were to describe, using a phrase, image, or metaphor, what it is like to be a girl/boy of your age these days, what would you write?”). With regards to demographic data, the research plan included age and gender as variable of interest since the study was exploratory and used a convenience sample. Data were collected anonymously, and all participants were briefed about the research aims and procedure. Participation in the study was on a voluntary basis, meaning that participants received no monetary or financial rewards. The study was approved by the Ethics Board at Milano-Bicocca University (prot. N. 0059806/21) and was conducted in keeping with the ethical principles laid down in the Declaration of Helsinki^29^ and the American Psychological Association code of conduct^30^. Informed consent was obtained from all participants and from parents for underage participants. During data collection (i.e., April to June 2021, a zoning policy was still in effect, based on the rate of contagion, while secondary school students were attending in-person classes 50% to 100% of the time, depending on the zone and the internal organization of schools.

### Measures

*World Health Organization Well-Being Index (WHO-5)* The five-item World Health Organization Well-Being Index (WHO-5) is a short rating scale measuring global subjective well-being^31^. The instrument has been used in many different settings to assess positive well-being and as a proxy for mental health^32^. The questionnaire items are: (1) ‘I have felt cheerful and in good spirits’, (2) ‘I have felt calm and relaxed’, (3) ‘I have felt active and vigorous’, (4) ‘I woke up feeling fresh and rested’ and (5) ‘My daily life has been filled with things that interest me’. Respondents rate each item on a Likert scale ranging from 5 (all of the time) to 0 (none of the time). The raw WHO-5 scores are computed by summing the scores for the individual items, yielding global scores ranging from 0 (no well-being) to 25 (maximal well-being) which are then conventionally converted to a scale of 0–100. A generally accepted threshold for poor well-being and the risk of developing depressive symptoms is less than 50^33^. In this study, Cronbach’s alpha reliability coefficient (α)^34^ was 0.817.

*Qualitative material* In line with our research aims, we analyzed the open-ended question “At this time, what are the times, situations, or events that help you feel good?” with a view to gathering direct information about how adolescents tried to taking care of their well-being during the COVID-19 health emergency. A total of 223 responses were collected, totaling 1915 words, with an average response length of 8.6 words. In terms of missing values, 6 participants did not respond or responded "I don't know," resulting in a missing value rate of around 2.5%.

### Data analysis strategy

We analyzed the data from our mixed-method questionnaire using quantitative textual analysis (QTA)^35^. QTA is a form of qualitative content analysis and assumes that (1) words that tend to appear together (i.e. close proximity) in a given context may be interpreted as related to a common lexical theme or concept within the discourse under study^36^ and (2) traditional statistical techniques may be used to analyze narrative data^37^. Hence, we analyzed the adolescents’ replies to the question “If you were to describe, using a phrase, image, or metaphor, what it is like to be a girl/boy of your age these days, what would you write?” via co-word analysis of correspondence based on our research interests (CA)^38^. The advantage of using CA to analyze this kind of material is that this method allows the researcher to examine the structure of a dataset by rescaling a set of proximity measures into visual distances representing specific locations in a spatial (Cartesian coordinate system) configuration^39^. The analysis yields word-maps which allow the researcher to identify recurring themes, their degree of salience, and how they relate to one another. We assessed similarities via the chi-square and Salton’s cosine indexes^40^ along with their statistical significance (set at p < 0.05). Salton’s cosine allows us to organize the relations geometrically so that they can be visualized as structural patterns of relations^41^.

To make the results of the co-word analysis more understandable, word-based concept mapping tools based on multivariate QTA methodologies may be used to identify dominant themes, their relative weight, and how they relate to one another within a given set of textual data^42^. Many studies in the field of health psychology and health promotion^43,44^ have suggested that common cluster analysis of textual data may be an interesting solution when researchers wish to gain meaningful insight into participants' words by bringing a positivist approach to bear on qualitative data^45^. In the present study, we used the k-means cluster analysis algorithm^46^. The k-means algorithm first groups objects into an arbitrary number of clusters, then computes cluster centroids and assigns each object in such a way that the squared error between it and the empirical mean of a cluster is minimized (i.e., Euclidean distance is used). K-means, like other techniques, seeks to minimize variability within clusters while maximizing variability between clusters^47^. A second critical issue in performing k-means cluster analysis in exploratory QTA is determining the optimal cluster configuration, where optimal refers to the outcome among all possible grouping combinations that presents the full set of the most meaningful associations^48^. Determining what distribution of clusters provides a better understanding of data requires the selection of an objective 'measure of optimal partitioning' (or clustering validity criteria). We chose Calinski- Harabasz index, which is also known as the Variance Ratio Criterion (VRC)^49^ from among the available measures because it evaluates the quality of data partitions according to a standard formula. Specifically, the greater the value of the between variance-within variance ratio normalized with respect to the number of clusters, the superior the data partition. To find the best configuration, we ran cluster analysis on the word co-occurrence matrix with varying numbers of clusters (from three to nine), choosing the solution with the best local VRC peak. For all analyses, the alpha level was set at 0.05. In the context of the present study, we expected that the output of the CU would allow us to analyze adolescents’ chosen metaphors by grouping “naturally” occurring emerging themes as a function of lexical similarity and in relation to well-being scores. All analyses were conducted using TLAB 5.0 and SPSS 21.0.

### Data cleaning and general descriptive statistics for the lexical corpus

As with other data exploration techniques, QTA required a pre-processing stage to prepare data for analysis. As recommended in the literature, we conducted *normalization* (removing all general function words such as articles, connection forms, and prepositions); *lemmatization* (reducing all inflected words to their root form as found in the dictionary) and *synonimization* (reducing words that may be considered equivalent from the semantic point of view—e.g., illness and sickness—ì to the same root form) with a view to preserving the accuracy of the textual data and preparing the database for running algorithms designed to generate both occurrence and co-occurrence matrices (for details about the process, see^50–52^).

The resulting qualitative database comprised 1616 occurrences, 652 raw forms, and 439 hapaxes (i.e., words that occurred only once in the text). By adopting a threshold of at least four occurrences (text coverage 83%), root type/token ratio (an index of text richness^53^) was 16.21, suggesting that the data were suitable for multiple correspondence analysis.

## Results

The results are divided into two sections. In the first section, we present quantitative data (i.e., descriptive statistics and zero-order correlations) concerning the levels of general well-being recorded in the adolescent sample using the World Health Organization threshold. In the second section, we summarize the results of the co-word correspondence analysis and subsequent clustering procedure.

The quantitative data outcomes are reported in Table 1.

**Table 1:** Summary of descriptive statistics and zero order correlations between demographic characteristics and levels of well-being.

	1	2	3
1. Gender	–		
2. Age	0.010	–	
3. WHO-5	− 0.228**	− 0.174**	–
Mean	–	16.64	52.61
Standard Deviation	–	1.46	22.25

*p < 0.01, **p < 0.001, *N* = 229.

**Figure 1:**
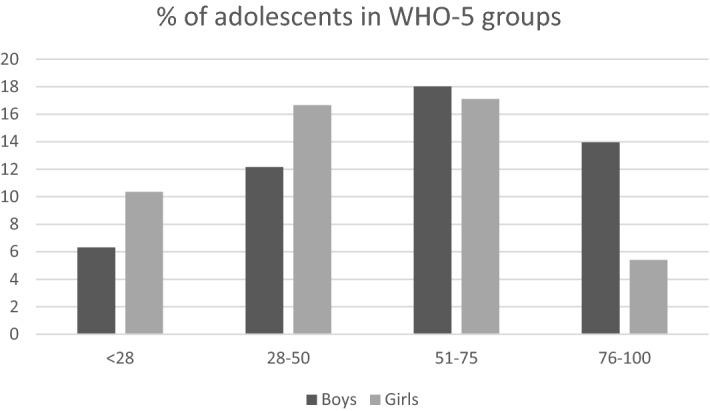
Adolescents grouped according WHO5 scores. Participants with scores of under 28 were at risk of clinical depression, while those with scores of over 75 displayed high levels of well-being.

The zero-order correlations suggest that the adolescents’ levels of well-being were negatively associated with age and gender, with younger participants reporting greater well-being and girls (WHO-5 mean score = 47.6) reporting less happiness than boys (WHO-5 mean score = 57.9). In this regard, analysis of variance revealed that the difference in levels of well-being between gender-based groups was statistically significant [t(1,220) = 3.54, p 0.001]. In addition, 36.6% of boys and 54.5% of girls obtained scores of less than 50, indicating that they were at risk of developing depressive symptoms. Furthermore, 12.5% of boys and 20.9% of girls scored less than 28, suggesting that they were at risk of clinical depression. Before moving on to the qualitative analyses, in Fig. 1 we show the percentages of boys and girls classified according to the World Health Organization’s well-being spectra. This figure illustrates the differences in well-being scores between boys and girls, particularly in the group at risk of developing clinical symptoms of depression and the group reporting high well-being.

The first result of the QTA concerned the words most frequently used by the cohort of adolescents to describe the moments, situations, or events that help them feel good during the COVID-19 syndemic. Given that frequently occurring words reflect recurring themes in a textual corpus and serve as the foundation for more complex coding categories, this is a preliminary form of analysis. The most frequently occurring words in the data set (with the number of occurrences reported in brackets) were: friends (137), family (51), to hang out (38), sport (18), music (18), boyfriend (16), to play (15), time (15), to meet (10), to chat (9), to watch (8), to listen (8), home (7), to help (7), people (6), on-line (5), to sleep (5) and alcohol (4). Even this initial look at the data provides some insight into the contents of adolescents’ strategies for coping with difficulties related to the syndemic; however, this level of interpretation is still quite biased (e.g., word frequency count is not weighted in relation to the length of responses), and it does not reveal the underlying structures in the data or the associations between words. When cluster analysis is used, it provides a more detailed picture. Because the evidence reviewed in the literature does not provide a theoretical framework for the structure of coping strategies, we began our exploratory analysis by determining the most appropriate cluster configuration for our qualitative data. Table 2 displays the values obtained for this purpose via the Calinski-Harabasz index.

**Table 2:** Selection of the number of clusters using the Calinski-Harabasz Index (VRC).

		3	4	5	6	7	8	9
S2B		0.568	0.897	0.938	1.012	1.113	1.184	1.173
S2W		0.694	0.412	0.349	0.301	0.264	0.232	0.209
VRC		88.3919	155.886	143.791	143.227	148.948	153.869	147.327

S2B, between-cluster variance; S2W, within-cluster variance.

**Figure 2:**
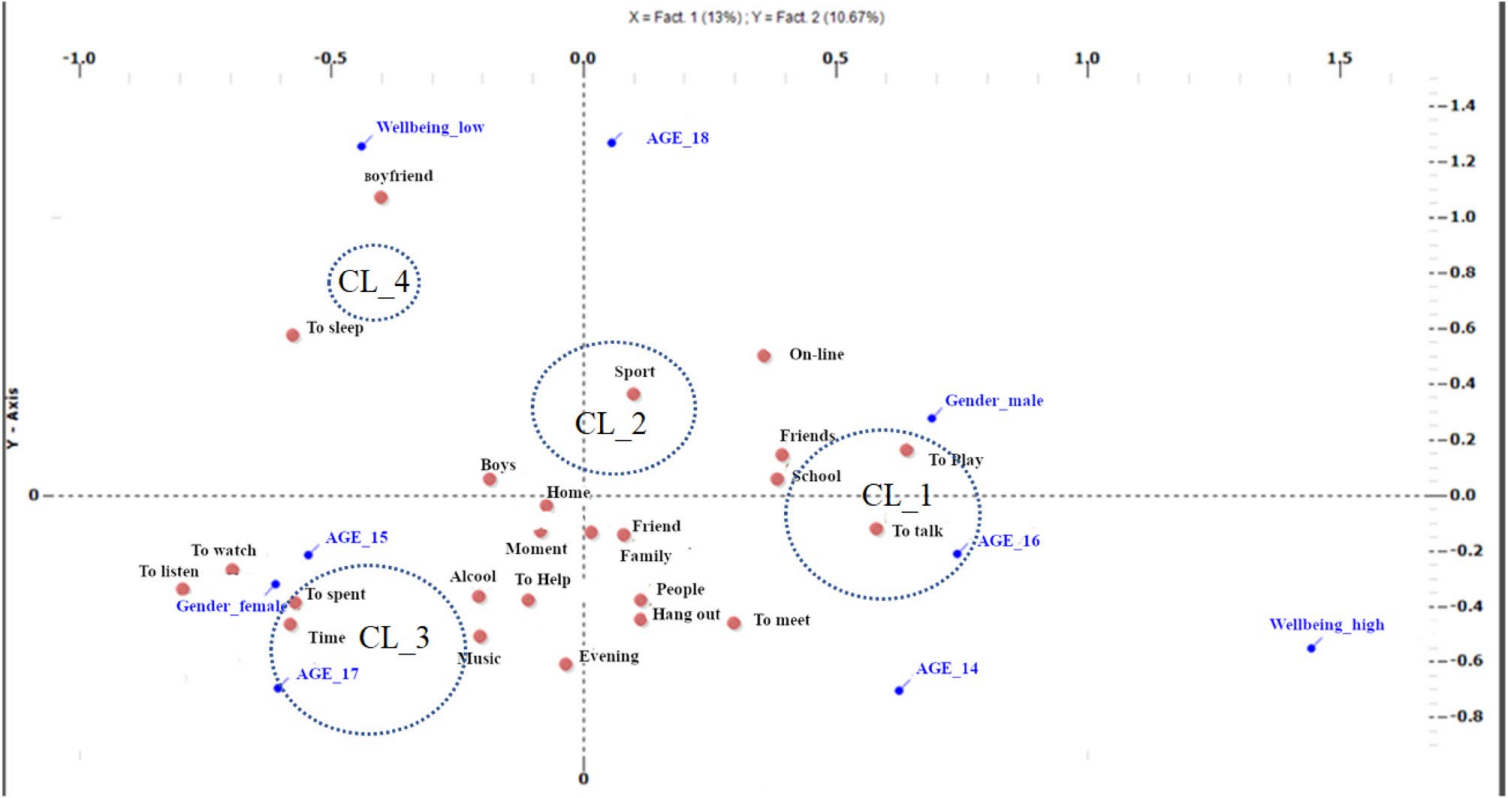
Graphical representation of clusters and occurrences in a Cartesian Space.

The VRC values revealed that, based on the defined word co-occurrence matrix, the optimal configuration was a solution with four distinct clusters. Peak VRC was found to explain 66.2% of total variance, with low within-variance values: cluster_1 (CL1, ssw = 0.162), cluster_2 (CL2, ssw = 0.131), cluster_3 (CL3, ssw = 0.081) and cluster_4 (CL4, ssw = 0.073). In terms of cluster density, the partition of words across the clusters was relatively even and satisfactory, with CL1 including 35.1% of replies and CL2, CL3 and CL4 including 22.8%, 31.9% and 10.1%, respectively. We then investigated the main contents of the coping strategies adopted by adolescents by calculating the association between the replies grouped in each cluster and the cluster itself (in terms of distance from the centroids). In addition, we evaluated the associations between variables (e.g. age, gender and levels of well-being) and clusters by calculating χ^2^ and its statistical significance. Finally, we plotted the cluster coordinates in two-dimensional factorial space to bring to light the meaning of the individual factors. Figure 2 offers a graphical representation of the four-cluster solution.

Cluster 1: In general, this cluster was associated with boys (χ^2^ = 6.35, p = 0.012) aged 16 years (χ^2^ = 5.85, p = 0.016) who reported a high level of well-being (χ^2^ = 6.31, p = 0.012). Quotes from this first cluster include: “*hanging out with friends, family, music*” (Boy, 16 y.o, WHO5 = 64), “*the rare evenings when I get together with my group of friends to quietly play some board games*” (Boy, 17 y.o., WHO5 = 72), “*being with my friends, going out, and talking to people close to me*.” (Girl, 16 y.o., WHO5 = 64) and “*being with my friends*” (Girl, 16 y.o., WHO5 = 72).

Cluster 2: This cluster grouped 18-year-old adolescents of (χ^2^ = 11.85, p = 0.001) with low levels of well-being (χ^2^ = 9.42, p = 0.002). Representative quotes included in the cluster were: “*Being with my family or boyfriend, or practicing sports*” (Girl, 18 y.o., WHO5 = 44), “*being with myself*” (Boy, 18 y.o., WHO5 = 38), “*Resting, going out, shopping*” (Male, 16 y.o., WHO5 = 24), “*I listen to music very often, in the evening I sometimes spend time on video calls with "friends" I met online who are from different countries*” (Male, 16.y.o, WHO5 = 36).

Cluster 3: this cluster was associated with 17-year-old adolescents (χ^2^ = 4.60, p = 0.032) with a medium–low level of well-being (χ^2^ = 4.11, p = 0.042). Quotes include: “*Nighttime, when all is silent and the thoughts screaming in the head fly away*” (Male, 19 y.o., WHO5 = 44), “*sport, alcohol, family*” (Male, 18 y.o., WHO5 = 40), “*Making music, being with my family, and going out for leisurely walks*” (Girl, 17 y.o., WHO5 = 54) and “*I do well in class, in the afternoon I never go out except sometimes with only one friend, so being in class with my classmates makes me feel good because there is no need for me to arrange to be with them*” (Male, 15 y.o., WHO5 = 48).

Cluster 4: The final cluster, which was the least dense accounting for approximately 10% of responses, was exclusively associated with “older” participants aged 19 years (χ^2^ = 16.18, p = 0.032). In this case, representative quotes included: “*Seeing my mother happy, feeling right with myself*” (Male, 19 y.o., WHO5 = 44), “*Dancing*” (Girl, 16 y.o., WHO5 = 60), “*Playing online games, watching anime, and talking with friends*” (Male, 17 y.o., WHO5 = 76) and “*taking advantage of my free time*” (Male, 18 y.o., WHO5 = 56).

Before proceeding with the last step in the data analysis, we deemed it of interest to list all the coping strategies deployed by the adolescents with a WHO-5 well-being score of under 28: “*leisure time”, “my family”, “no one”, “Being with friends”, “listening to music and playing with the Xbox”, “seeing my friends outside of school”, “seeing friends and sleeping”, “Seeing my friends and playing football with my team”, “being with friends”, “spending time with my friends”, “there is no time”, “going to school”, “I have no idea”, “sleeping and eating” and “resting, going out, shopping*”. With a view to comparison, we similarly listed all the coping strategies drawn on by the adolescents with a WHO-5 well-being score of over 75: “*My friends, soccer, and my girlfriend's love”, “My family and my girlfriend”, “Family and friends”, “Being with friends”, “Being with family during the holidays”, “My parents' affection and my friends' trust”, “When I am with friends, when I am with my family at home or outside”, ”hanging out with friends”, “being with the people who make me happy, friends and family”, “hanging out with friends and family”, “family, hanging out with friends and playing soccer”, “soccer”, “sports, hanging out with friends and playing” and “Being with people who love me”.* It should be noted here that there are substantial differences in the well-being strategies described by these two groups of adolescents (high vs. low well-being). In the descriptions of the high well-being group, for example, both the actions taken to feel better and the different social actors (friends, family members, boyfriends) involved, as well as the positive emotions and feelings felt (love, affection, happiness), were also present. This component (positive emotion and feelings) was lacking in the descriptions of the group with low well-being.

The final step in our data analysis was to label the two axes of Cartesian space with a view to defining a framework of meaning within which to organize the clusters. The principal axis is the straight line that runs closest to the profile point and passes through the zero point, hence meaning is identified first along the *y*-axis and then along the *x*-axis (Fig. 2). Conventionally, this type of graph in QTA cluster analysis is interpreted in terms of the geometric figures that can be drawn between the representation’s outermost points (see^35^): in this case, the “triangle” drawn between CL1 (center-right), CL4 (top-left and bottom-center), CL3 (bottom-left). Looking at the first axis (X), we can see that this dimension has two poles: the negative extreme to the left is constituted by CL4 and CL3, whereas the positive extreme to the right is CL1 (the terms positive and negative are only artifacts of the calculus process, and they could easily be inverted within this framework). CL1 was associated with high levels of well-being, while CL4 and CL3, at the opposite pole, grouped adolescents reporting low or medium–low levels of well-being. Consequently, the *x* dimension may be labeled level of well-being. Similarly, the second axis (*y*) divides CL4 at the positive extreme from CL3 at the negative extreme, with CL2 and CL1 remaining in the middle. CL4 (and CL2, whose projection on the Cartesian axis is very close to that of CL4) included coping strategies that may be conducted alone or with a limited number of people, such as: dancing, playing online games, shopping, and making music. On the other side, CL3 (and CL1, whose projection on the Cartesian axis resembles that of CL2) seemed to group coping strategies that were more social and collective in nature, such as: spending time with family and friends. Hence, the *y*-axis may be labeled as a second dimension of coping strategies that reflects a notion of I-ness as opposed to a sense of We-ness^54^.

To summarize our findings from this QTA of qualitative data collected from adolescents during the COVID-19 syndemic and integrate them into the existing framework of coping strategies for well-being, the cluster analysis results implies the existence of two 'macro-dimensions' that allow us to organize otherwise apparently “atomized” elements of subjective experience during a time of health emergencies and existential uncertainty. A first factor termed level of well-being and a second termed i-ness/we-ness. In this sense, coping strategies of adolescent during COVID-19 syndemic seem not only to range from individuation (I-ness) to affiliation (we-ness)—or, to draw on the words of Wiekens and Stapel, from a sense of togetherness (We-ness) to a sense of separateness (I-ness); rather, they also seem to be strongly associated with different levels of well-being.

## Discussion

The aim of the present study was to advance our understanding of adolescents’ perceived personal well-being during the COVID-19 public health emergency. Besides assessing participants’ levels of well-being by means of a validated and standardized instrument such as the WHO-5, we were interested in exploring the way adolescents were taking care of their mental health during the syndemic period. Our results were generally in line with the hypotheses that we had formulated, offering insights into how best to support adolescents’ mental health trajectories, informing a complex interpretation of the concept of wellbeing, and calling for further, more in-depth investigation.

### The role of age and gender in adolescents’ wellbeing

Looking at the results for gender and age differences in relation to perceived well-being, our data support the findings already reported in the literature: girls perceive significantly lower levels of well-being than their male peers^55,56^. In addition, older ages are correlated with lower levels of well-being with a general decline in mental well-being with increasing age, whereby older adolescents experience lower levels of life satisfaction, are less likely to report excellent health, and suffer more frequent mental health problems^57^. Furthermore, the same study showed that, by age 15, girls report poorer mental well-being than boys. The COVID-19 syndemic has confirmed and in some cases accentuated these differences, with older female adolescents suffering more from anxiety and depressive symptoms^58^. This situation is part of a broader picture whereby adolescent mental health has been undergoing a general decline in recent years^59^: for example, a study^60^ identified, from 2018 to 2020, a decrease in mean perceived well-being, as measured by the WHO-5, from 43.7 to 35.8 (albeit that both of these scores invite reflection on the state of mental health in adolescence more generally). Thus, it seems that the COVID-19 emergency has accelerated a process that had already been underway for some years, and which requires policy makers to urgently examine the adequacy of current mental health promotion services and practices.

### Self-care practices

The results of the QTA offer us a more in-depth and nuanced understanding of the conditions that influence adolescents’ well-being, including the role of gender and age. If we examine cluster 1, we find mainly younger male adolescents, who, when asked the open-ended question about how they take care of their personal well-being, answer by naming strategies chiefly aimed at maintaining peer relationships and teams sports-playing. This cluster is associated with high levels of well-being. On the other hand, in clusters where levels of well-being are lower, adolescents refer to the use of more individualistic and intimate, but also more "passive" strategies (music, shopping…). When looking at the coping strategies reported by those with "extreme" scores on the well-being curve (under 28 and over 75), key differences emerge. Adolescents with scores of < 28 (who are thus potentially at risk of depression) report seeking support from relationships, yet positive affective states rarely feature in their responses, and they make greater use of "static" verbs ("I'm with," "I see…"). Furthermore, in the responses of the group of adolescents with very low levels of well-being, food or alcohol intake (about 5%) also appeared as—dysfunctional—coping strategies, along with higher levels of apathy ("sleep longer"…). In contrast, adolescents with high levels of perceived well-being reported actively seeking out social support and positive relationships, within their families and among their friends, and these efforts were more frequently and explicitly associated with affective and emotional states. This finding corroborates studies in the literature which suggest that adolescents’ growing desire for autonomy and independence from parents and to belonging to a peer group^61,62^ takes shape in parallel with the maintenance of close and positive relationships with family as a key requirement for psychological well-being and adjustment^63^. In particular, adolescents who have poorer and dysfunctional family interactions and relationships experience greater psychological maladjustment. In the context of the public health emergency, everyday living conditions, especially the fact of sharing the same spaces with family for a prolonged length of time, likely amplified the impact of positive vs dysfunctional family relationships on levels of well-being. We might speculate that the participants in the present study who most frequently mentioned their family as a positive resource are those who were already embedded in more protective and functional systems. Nevertheless, examining the levels of well-being of clusters 1 and 2 (medium–high) versus cluster 3 (medium–low), it seems that it is the combination of family-friends (as in cluster 1 and 2) as sources of support, as opposed to "just" family (as in cluster 3), that makes the difference with respect to levels of perceived well-being.

When the dimensions of well-being and coping strategies are jointly represented along Cartesian axes, a continuum emerges from high levels of well-being associated with more affiliative strategies (we-ness/togetherness) to low levels of well-being associated with more individualistic strategies (I-ness/separatedness). Again, the collective dimension emerges as a resource. However, this result prompts reflection about access to social support as a strategy for coping with situational stress. Thinking "we are all on the same sea" is a view that may help, but it is also true that "everyone has a different boat": those who experience positive and satisfying family and extra-familial relationships may be more likely to identify and seek out the collective dimension as a potential source of protection against stress, while those who had already been experiencing conditions of marginality or dysfunctional family relationships or vulnerability prior to the advent of COVID-19 may find it more difficult and/or unhelpful to turn to more "social" coping strategies. We might reflect on how much this syndemic has widened such gaps, which are not only economic but also social and political, among people in general and among adolescents in particular. Studies have proven that in fragile adolescents (suffering from anxiety and depression), the impact of the public health emergency has further exacerbated their situation and increased the distance between these youths and peers with good levels of mental health who are well integrated at the socio-relational level: indeed, the literature shows that adolescents who experience greater symptoms of anxiety and depression experience a deterioration in social well-being over time, and receive less social support and greater victimization from peers^64^. Other similar studies have shown how the COVID-19 outbreak and the related risk-reduction strategies have changed the social contexts of adolescents in low- and middle-income countries, with profound implications for their well-being, especially in the case of vulnerable adolescents, including those affected by poverty and armed conflict. In such contexts, pre-existing conditions of disadvantage have had a negative impact on the mental health of adolescents, especially that of girls^65^.

Thus, in the current syndemic setting, the issue of "non-communicable disease" emerges strongly, coupled with, and exacerbating the impact of the virus on the physical health of self and significant others. In this scenario, it is not difficult to discern whether the increase in mental health symptoms is the result of the disease itself (directly or indirectly experienced, or as a source of concern for one’s own safety) or the related restrictive measures (e.g., separation from friends, disruption of school, etc.). In any case, it is crucial to carefully weigh the potential benefits of reduced COVID-19 transmission against the detrimental effects on mental health of social isolation, especially in adolescents. From this perspective, insistent calls for social distancing from the authorities seem unfair and counterproductive: while physical distancing may offer protection from a physical health perspective, we need social closeness to maintain good mental health.

### Limitations

This study, like others of its kind, features several limitations that should be noted. First, the study is cross-sectional, which means that the teenagers were questioned at a point in time when the COVID-19 epidemic was still ongoing. While this was congruent with the research aims, the research design offers no information about the dynamic evolution of the phenomena under observation, either in terms of well-being or in terms of self-care strategies. A second limitation concerns the fact that the interviews were administered online. Although this approach facilitated data collection at a time when mobility constraints and public health measures made it difficult to gather data directly in the field, it raises concerns regarding the sample's effective representativeness. The most susceptible groups of teenagers or those with educational and economic difficulties may have had restricted access to the Internet and computer technologies, affecting their ability to respond the survey. This means that caution is required in generalizing our findings to all Italian teenagers. Another limitation is that the research plan only considered demographic variables such as age and gender. Instead, studies are needed to detect the effects of other contextual variables that may be associated with adolescent well-being in order to better assess the dynamics of syndemics. In the future, follow-up studies within this line of inquiry should be conducted with larger samples and longitudinal designs, in order to gain a clearer picture of the variables studied, in terms of both the stability of the identified associations and the scope for change, especially given the fact that adolescence in general is a period of transition and rapid transformation.

## Data Availability

The datasets generated during and/or analysed during the current study are available from the corresponding author on reasonable request.
